# The Diagnostic Value of Brush Cytology Alone and in Combination with Tumor Markers in Pancreaticobiliary Strictures

**DOI:** 10.1155/2015/580254

**Published:** 2015-03-26

**Authors:** Ufuk Barış Kuzu, Bülent Ödemiş, Nesrin Turhan, Erkan Parlak, Selçuk Dişibeyaz, Nuretdin Suna, Erkin Öztaş, Muhammet Yener Akpınar, Adem Aksoy, Serkan Torun, Hakan Yıldız, Ertuğrul Kayaçetin

**Affiliations:** ^1^Department of Gastroenterology, Turkiye Yuksek Ihtisas Education and Research Hospital, Kiziliay Street No. 4, Sihhiye, 06100 Ankara, Turkey; ^2^Department of Pathology, Turkiye Yuksek Ihtisas Education and Research Hospital, Kiziliay Street No. 4, Sihhiye, 06100 Ankara, Turkey

## Abstract

*Aim*. Differentiation of malignant and benign strictures constitutes a problem despite the increasing experience of the endoscopists, radiologists, and pathologists. The aim of our study is to determine the factors that affect the efficacy of the ERCP guided brush cytology in PBS and to evaluate its diagnostic success when used alone and together with tumor markers. *Method*. The data from brush cytologies of 301 PBS patients were collected retrospectively and analyzed. The final diagnosis was approved based on the histological examination of the tissue taken surgically or by other methods. In the absence of a histological diagnosis, the final diagnosis was based on radiological studies or the results of a 12-month clinical follow up. *Results*. A total of 28 patients were excluded from the study. From the remaining 273 patients 299 samples were analyzed. The sensitivity and the specificity of brush cytology in diagnosing malignancy are 62.4% and 97.7, respectively. The sensitivity of brush cytology increased to 94.1% when combined with CA-19.9 and CA-125. *Conclusion*. Brush cytology is a useful method in diagnosing pancreaticobiliary strictures. Advanced age, stricture dilatation before sampling, the presence of a mass identified by radiological studies, high levels of CA-19.9, ALT, and total bilirubin increase the sensitivity of brush cytology.

## 1. Introduction

Pancreaticobiliary strictures (PBS) can be a result of most commonly inflammatory and neoplastic diseases of the pancreas, bile ducts, gallbladder, and ampulla of Vater. Benign strictures can be treated with conservative methods such as endoscopic or percutaneous stent placement and dilatation. The treatment method preferred for malignant strictures is surgical excision. In unresectable cases palliative treatment is offered [1–3].

The most important step in patients with radiologically identified biliary stricture is the differentiation of benign from malignant strictures. The importance of preoperative definite diagnosis using noninvasive methods in PBS patients is increasing due to the introduction of new neoadjuvant treatments. However, the differentiation of malignant and benign strictures constitutes a problem despite the increasing experience of the endoscopists, radiologists, and pathologists. Since biliary and pancreatic canal lesions cannot be accessed easily for biopsy, cytological techniques have become the first diagnostic method in most cases [4–8]. The available sampling techniques for cytopathological evaluation of the bile ducts are intraductal bile aspiration cytology, endobiliary forceps biopsy, cytopathological analyses of plastic bile stents, brush cytology, and fine needle aspiration biopsy (FNAB) [9]. Even though, percutaneous radiologically guided FNAB seems like an accurate method, it is dependent on the operator and requires an evident lesion for sampling. At the same time, the seeding of the cancer cells in the needle tract constitutes a problem for patients who are candidates for surgery [10]. Endoscopic retrograde cholangiopancreatography (ERCP) assisted brush cytology can be performed easily, is safe, and is used widely. These characteristics make ERCP the most commonly used method for tissue sampling [6–8, 11, 12].

The aim of our study is the determination of the factors that affect the efficacy of ERCP assisted brush cytology in PBS and the evaluation of its diagnostic success when used alone and together with tumor markers.

## 2. Methods

### 2.1. Study Design and Patients

This study was conducted in the ERCP unit of the Turkiye Yuksek Ihtisas Education and Research Hospital, a third level reference hospital, where more than 2000 ERCP procedures are performed annually. The data from 11785 patients, diagnosed with PBS and whose brush cytologies were taken, were selected from all the ERCP patients between January 2008 and June 2013. In this time period, the data from 301 patients were analysed retrospectively.

### 2.2. Brush Sampling Procedure and Technique

All the procedures were performed by 3 experienced pancreatobiliary endoscopists using an Olympus video duodenoscope (Olympus TJF 240 or JF 240, Tokyo, Japan). The cytological samples were taken in similar ways in all the cases. After pancreaticobiliary cannulation, contrast material diluted with saline in a ratio of 1/2 was injected to identify the localization and the length of the stricture. After that a guide wire (Jagwire 0.035 inch, Boston Scientific, USA) was introduced inside the duct. In some cases, the stricture was dilated using a 7–10 FR dilatation catheter or a 6–8 mm dilatation balloon before sampling. After that, the double lumen cytology brush (RX-cytology brush; Natick, MA) was moved forward and backward 10–15 times to get the sample. The samples obtained with the brush were spread over 4 glass slides, fixed, and stained with May-Grünwald Giemsa in the pathology laboratory. Additionally, the end portion of the brush was cut and put into formalin solution for the cytological examination of the crumb tissue on the brush.

### 2.3. Study Variables and Cytopathological Examination

The demographic characteristics, cytopathological results and imaging studies reports (at most 1 month before the procedure), and clinical data were collected for each patient in the same way. The data were taken from the Dataselin formation systems, Ankara, Turkey. The AviCenna Hospital Information Management supports worldwide accepted standards (ICD-10, SNOMED, ATC, DMDN, etc.). The patients who had stricture dilatation before the sampling were recorded. Additionally, the serum biochemical parameters and the tumor markers were recorded before the first sampling (at most within 1 month). Biochemical parameters included glucose (80–110 mg/dL), total bilirubin (<1.2 mg/dL), aspartate aminotransferase (AST) (<33 U/L), alanine aminotransferase (ALT) (<33 U/L), alkaline phosphatase (ALP) (33–105 U/L), and gamma glutamyl transferase (GGT) (5–36 U/L). Tumor markers included carcinoembryonic antigen (CEA) (0–3 ng/mL), alpha-fetoprotein (AFP) (0–7,4 IU/mL), carbohydrate antigen-19.9 (CA-19.9) (0–35 U/mL), and carbohydrate antigen-125 (CA-125) (0–35 U/mL). Serum levels of these markers could be obtained form 238, 181, 241, and 189 patients, respectively.

The patients were classified into 4 groups according to well accepted and widely used criteria [13, 14]: (1) benign, (2) containing reactive atypical cells, (3) suspicious of malignancy, and (4) malignant. Combination of groups with malignancy and suspicious of malignancy was considered to be positive result, whereas combination of benign and group with reactive atypical cells was considered to be negative result.

The patients were classified into 4 groups according to the location of PBS: intrahepatic bile ducts, perihilar region (hilus and common hepatic duct), common bile duct, and pancreatic canal.

### 2.4. Final Diagnosis (Study Criterion Standard)

Patients with a definite diagnosis of a benign or malignant stricture were included into the study. The final diagnosis was put based on the histological examination of the tissue samples obtained surgically or via other methods. In the absence of a histological diagnosis, the final diagnosis was put via radiological studies or depending on the result of a 12-month clinical follow-up period. Patients not meeting the follow-up gold standards, having a nondiagnostic cytology (insufficient material) result, and who are with a tumor that can be observed endoscopically in the papillary region were excluded from the study.

### 2.5. Statistical Analysis

The data were analyzed using the Statistical Package for Social Sciences (SPSS) program version 18 (SPSS Inc., Chicago, IL, USA). Continuous variables were expressed as mean (±standard deviation) and, if necessary, median value (the distance between quarters; 25–75 percentiles), whereas categorical variables were expressed as frequency and percentages. Student's *t*-test was used to compare groups of continuous variables, whereas Pearson Chi-square test, Fisher test, and definitive Chi-square test were used to compare groups in terms of categorical variables. The previously identified factors associated with positive brush cytology results were evaluated using multivariable logistic regression analysis. The Hosmer-Lemeshow goodness of fit test was used to assess the fit of the models. The diagnostic role of tumor markers in predicting positive brush cytology was assessed using receiver-operating characteristic (ROC) curve analysis. In cases where significant cut off values were obtained sensitivity, specificity, positive predictive value (PPV), negative predictive value (NPV), and accuracy ratio were calculated. Values where type 1 error level was less than 5% were considered to be statistically significant.

## 3. Results

Three hundred and one patients participated in our study. Twenty-eight patients were excluded from the study (14 patient had unknown final diagnoses, 10 patient's brush cytology results were insufficient, and 4 patients had papillary tumor that can be seen endoscopically). From the remaining 273 patients 299 samples were analyzed. The average age of the patients was 61.2 (22–90) with 53.5% of the subjects being female (Table 1). The most common stricture location was common bile duct (*n* = 166). The most common cause of benign strictures was biliary stones (*n* = 57).

**Figure 1:**
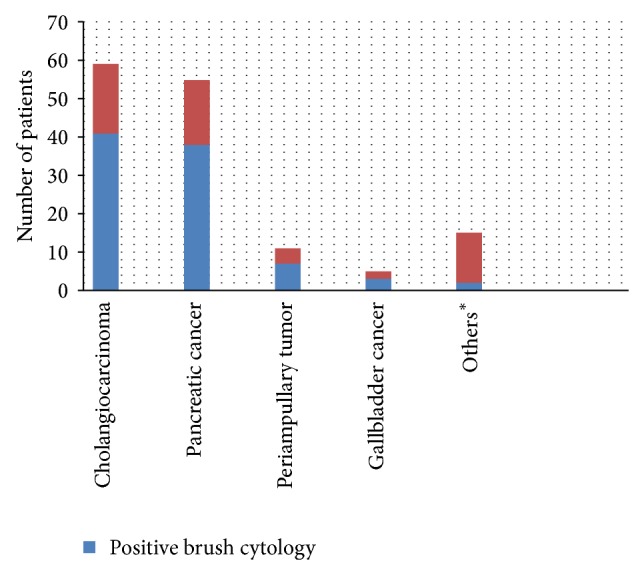
Distribution of the positive biliary brush cytology in malignant strictures. ^*^Intraductal papillary mucinous neoplasm, hepatocellular carcinoma, and metastasis.

In the final diagnosis, 141 patients (51.6%) had malignant strictures, whereas 132 patients (48.4%) had benign strictures. In our study, the brush cytology was positive in 91 patients (33.3%) and negative in 182 patients (66.7%). Eighty-eight of the patients with malignant strictures (62.4%) were diagnosed with brush cytology, whereas the remaining 53 patients were diagnosed using FNAB, surgery, radiological imaging, and clinical followup. The most commonly detected neoplasm was cholangiocarcinoma (CCA), followed by pancreas adenocarcinoma, periampullary tumor, and gallbladder tumor. Brush cytology detected 41 out of 59 patients with CCA and 38 out of 55 patients with pancreatic cancer (Figure 1).

In our series, there were 88 true positive, 3 false positive, 129 true negative, and 53 false negative cytology results. The specificity of brush cytology in diagnosing malignancy is quite high (97.7%), whereas its sensitivity is partially low (62.4%) (Table 2). The sensitivity was observed to be decreased through the biliary canal from proximal part to distal. However, sensitivity and specificity of brush cytology between pancreatic and biliary canal and among specific parts of the biliary canal were not statistically different (Table 3).

Three patients had a false positive result. One of the patients was diagnosed with a benign stricture after Whipple operation, whereas the other two patients were diagnosed with benign strictures after repeated brush cytologies and biopsies from the periampullar region.

Twenty-six patients with negative cytology results had another brush cytology during the follow-up period due to clinical suspicion of malignancy. The cytology results of 20 patients did not change, whereas 6 patients were diagnosed with malignancy (2 pancreas adenocarcinoma and 4 CCA) in the second or third brush sampling. The sensitivity of brush cytology increased to 66.6% after adding the results of the second brush cytologies to the first ones.

Patients with positive and negative brush cytology results were compared in terms of age, gender, dilatation before sampling, size of the mass detected by CT/USG, stricture location, laboratory findings, and tumor markers. Statistical difference was present between the two groups in all variables, except for glucose, AFP, and stricture location. However, multivariate regression analysis revealed that among the above mentioned variables only advanced age: 1.02 (1.002–1.04), sampling before dilatation: 3.03 (1.5–6.07), mass larger than 10 mm detected by CT/USG: 1.7 (1.2–5.7), CA-19.9 level: 1.002 (1.001-1.002), ALT: 1.004 (1.001–1.007), and total bilirubin level: 1.11 (1.04–1.19) were independent predictors for positive brush cytology (Table 4).

**Figure 2:**
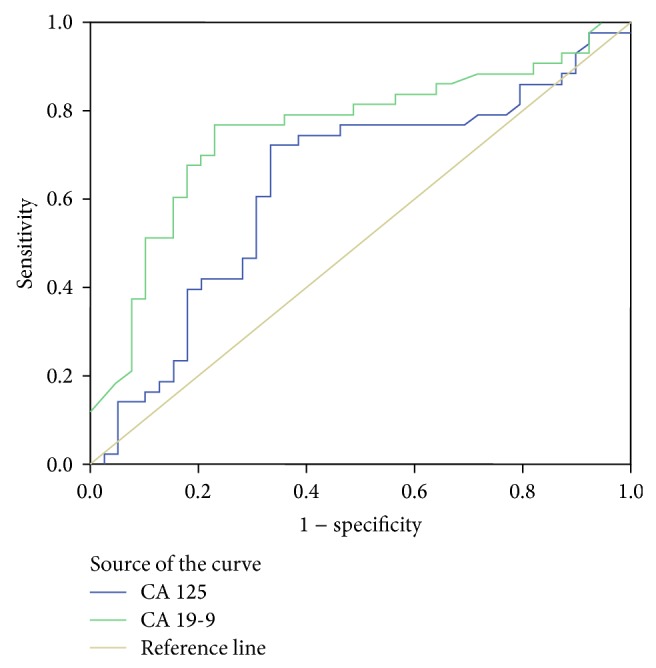
ROC curve for CA-19.9 and CA-125. The area under the curve (AUC) for CA19-9 is 75.4 (95% CI: 64.5–86.3). The AUC for CA-125 is 63.3 (95% CI: 50.9–75.7).

As shown in Table 5, serum levels of tumor markers in patients with malignant strictures are statistically higher. However, ROC curve analysis revealed that only CA-19.9 and CA-125 were significant in diagnosing malignancy. The optimal cut off values for these two markers were calculated as 72.5 U/mL and 17.5 ng/mL, respectively (Figure 2). At these cut off values, the sensitivity, specificity, and diagnostic accuracy of CA-19.9 and CA-125 were 73.8% and 79.5%, 76.5%, and 74.4% and 61.5% and 68.2%, respectively. The sensitivity of brush cytology increased to 94.1% when combined with CA-19.9 and CA-125, whereas its accuracy rate increased to 80.6% (Table 2).

## 4. Discussion

PBS is commonly encountered during ERCP procedures. Brush cytology, which is commonly used for diagnosing malignancies of the pancreaticobiliary was first described in 1975 by Osnes et al. [15].

In our study, 299 samples from 273 PBS patients were evaluated and their consistency with histopathological, clinical, and radiological findings was assessed. Compared to the data available in the literature, this study is one of the most comprehensive studies investigating the success of brush cytology in PBS patients. It is one of the rare studies evaluating the diagnostic success of brush cytology and tumor markers together in the identification of malignant strictures.

The specificity of brush cytology has been reported to vary between 95% and 100% [13, 16–22] and we calculated a specificity of 97.7% which is consistent with the results of previous studies. Three patients, two chronic pancreatitis and 1 sclerosing cholangitis patient, had false positive results. Stewart et al. [20] reported having 3 patients with false positive results; one was diagnosed with PSC, one with common bile duct stone, and one with chronic pancreatitis. The specificity of brush cytology in this study was found to be 98.1%. Another study that enrolled 180 patients reported having one patient, diagnosed with PSC, with false positive results, and the brush cytology specificity was reported as 98.1% [22]. False positive results were related to incorrect assessment of low grade dysplasia and reactive atypia in diseases that lead to benign strictures such as postoperative stricture, choledochal stone, PSC, or chronic pancreatitis.

In our study, the sensitivity of brush cytology was moderate (62.4%), but compared to the literature it was quite high. Previously published paper reported lower sensitivity of brush cytology in detecting pancreatobiliary malignancies than our study (35–48%) [23–28]. However, some studies reported higher diagnostic sensitivity of brush cytology (57–83%) [19, 29–31]. The reason for these differences in sensitivity has not been well understood. However, these differences may be accounted for by the experience of the pathologist, the technique of collecting the samples, and the different categorization of the cytological results (e.g., the exclusion of insufficient material or the classification of the suspicious samples as positive results). When the repeated brush cytology results were taken into consideration, the sensitivity of brush cytology increased to 66.6%. As in our study, other studies also reported an increase in the sensitivity of brush cytology after repeating the procedure. This is why repetition of brush cytology is recommended in cases of unexplained PBS [19, 22, 32].

Few studies investigating the effect of the tumor type and stricture location on the diagnostic success of brush cytology are available. In our study, stricture location was found to have no effect on the diagnostic success of brush cytology. Similar results were reported [5, 31], although there are some studies that reportthe opposite [33, 34]. As reported that the type of tumor affects the success of brush cytology [6, 7, 33], Kurzawinski et al. [7] demonstrated that the sensitivity of brush cytology is the highest for periampullar tumors, moderate for CCA, and the lowest for pancreas cancers. However, our results were inconsistent with these ones. In our study, the sensitivity of brush cytology for pancreas adenocarcinoma and CCA was almost at the same level (69.1% and 69.5%, resp.). The difference may be related to the formation of the stricture due to external pressure on the ducts and the inability to obtain enough cells for cytological analysis in case of pancreas cancer. In our study, 68% of the patients diagnosed with pancreas cancer were at an advanced stage. The reason why our rates were close to each other may be because the pancreatic tumors invaded the ducts and allowed collecting adequate amount of cells for the diagnosis. However, we noticed that brush cytology sensitivity in diagnosing metastasis, HCC, intraductal papillary mucinous neoplasm (IPMN) was low. Similar results have been reported also by other studies [35, 36]. The reason for this low sensitivity may be due to the external compression without invasion of the bile ducts, due to inability to collect enough cells because the biliary stricture is caused by submucosal growth, or due to the inability of the pathologists to recognize some tumor types, such as IPMN.

Our study is also the most comprehensive study investigating the factors affecting the efficacy of brush cytology. Using logistic analyses, only advanced age, CT/USG identified mass size, dilatation before sampling, ALT, CA-19.9, and total bilirubin levels were found to be independent predictors for positive brush cytology.

Previous studies have shown that dilatation before sampling increased the sensitivity of brush cytology [37, 38]. Similarly, we also found that dilatation before sampling increased sensitivity by 3-fold. Mohandas et al. [38] in their study calculated the sensitivity of brush cytology in detecting malignant strictures in cases done with and without dilatation as 63.3% and 26.6%, respectively (*P* < 0.003). Theoretically, the reason for this increase is thought to be related to the increase in the number of cells available for cytological evaluation after dilatation [35]. Using the same principle, Parasher and Huibregtse [39] used a new technique to excavate the stricture and noticed that the sensitivity of brush cytology in detecting malignancy increased to 100%.

Our study has shown that, also advanced age and/or identifying the mass via CT or USG increase the sensitivity of the brush cytology as reported in previous studies [22, 27].

Different from other studies, our study observed that CA-19.9 and ALT levels can be used as positive predictors despite having a minimal effect on positive brush cytology. In our population, for both laboratory findings, a 10-unit increase raised the possibility of a positive cytology by 2% and 4%, respectively. At the same time, we determined that every 5-unit increase in total bilirubin raises the possibility of positive brush cytology by 5.5-fold. This relationship has been reported also by other studies [27]. But our study strongly confirmed that total bilirubin level is an important predictor of positive brush cytology. It is not clear how these independent predictors increase the possibility of positive brush cytology. However, high levels of CA-19.9, ALT and total bilirubin, and the presence of radiologically detected mass indicate an advanced stage malignancy or a severe stricture.

Another aim of our study was the evaluation of the diagnostic accuracy of tumor markers alone or in combination with brush cytology in detecting malignant strictures. ROC curve analysis revealed that only CA-19.9 and CA-125 were significant in differentiating benign from malignant PBS.

CA-19.9 serum concentration increases in both malignant and benign disorders of the bile ducts [40–42]. This is why studies have reported different optimal values of CA-19.9 and its sensitivity in detecting malignancy was found to be 69–93%, whereas its specificity was reported as 78–98.5% [40–45]. In our study, the optimal value for CA-19.9 was calculated as 72.5 U/mL and its sensitivity and specificity were found to be 73.8% and 79.5%, respectively. Morris-Stiff et al. [40] determined the cut off value for CA-19.9 as 70.5 U/mL and its sensitivity and specificity as 82.1% and 85%. İt is suggested that CA-19.9, at its optimal cut off value can be a good predictor in the diagnosis of the tumors of the pancreaticobiliary region [40, 46].

CA-125 can be used in the pancreaticobiliary region malignancies [47, 48]. Compared to CA-19.9, studies regarding CA-125 are more limited and are more concerned with specific tumors of the pancreaticobiliary region. The literature reported sensitivity and specificity of CA-125 in diagnosing pancreatic cancers as 45.0–61.0% and 83.3%, respectively [43, 49, 50]. The sensitivity of CA-125 in detecting CCA has been reported as 75.7% [48]. The same study found that CA-125 was affected less by benign diseases of the pancreaticobiliary region compared to CA-19.9 and CEA [48]. In our population the cut off value for CA-125 was determined as 17.5 ng/mL. Its sensitivity and specificity in identifying pancreaticobiliary malignancies were found to be 74.4% and 61.5%, respectively.

In our study, brush cytology was found to have a moderate sensitivity and a high specificity, whereas CA-19.9 and CA-125 were shown to have high sensitivity and a moderate specificity. Brush cytology when combined with CA-19.9 and CA-125 achieved a high sensitivity in the differentiation of malignant and benign PBS (94.1%), whereas the specificity and the accuracy ratio for this combination was calculated as 54.5% and 80.6%. There are no other studies evaluating this combination in the diagnosis of PBS. In one study, combination of CA-19.9 and radiological studies had a sensitivity and specificity of 97.2% and 88.7%, respectively [40]. In another study, the combination of brush cytology with DNA analysis, CEA and CA-19.9 (cut off value for CEA and CA-19.9; 5 ng/mL and 100 U/mL, resp.) increased sensitivity, specificity, and accuracy values to 88%, 80%, and 84% [44].

Even though all the data were obtained from a valuable hospital information system in an objective manner, being a retrospective study has led to some limitations. This is the reason why the laboratory results of some patients could not be obtained and patients not meeting the gold standards were excluded from the study.

According to the results of our study, brush cytology, apart from being a simple and reliable method, is also an efficient modality for diagnosing PBS. Advanced age, the dilatation of the stricture before sampling, presence of a radiologically identified mass, total bilirubin levels, and high levels of CA-19.9, and ALT increase the sensitivity of brush cytology. The combination of brush cytology with CA-19.9 and CA-125 in PBS is useful. However, the absence of a defined optimal ranges, its unidentified costs and being affected by benign diseases of the pancreaticobiliary tract or other malignancies limit the use of tumor markers in the evaluation of PBS. Multicenter studies need to be conducted to assess the efficacy of different approaches in evaluating PBS.

## Figures and Tables

**Table 1:** Baseline characteristics.

Clinical characteristics	Value
Patient	273
Age (year)	61.2 ± 13.8
Male/female	127 (46.5)/(53.5) 146
Clinical diagnosis	
Malignant	141 (51.6%)
Cholangiocarcinoma	59 (41.9%)
Pancreatic cancer	55 (39.1%)
Periampullary tumor	7 (5.0%)
Gallbladder cancer	5 (3.5%)
Intraductal papillary mucinous neoplasm	4 (2.8%)
Hepatocellular carcinoma	3 (2.1%)
Neuroendocrine tumor	2 (1.4%)
Metastasis^*^	6 (4.2%)
Benign	132 (48.4%)
Common bile duct stone	57 (43.2%)
Postoperative stricture	25 (18.9%)
Primary slerosing cholangitis	22 (16.7%)
Chronic pancreatitis	21 (15.9%)
Other^**^	7 (5.3%)
Lesion location (malignant/benign)	
Intrahepatic	4 (2.8%)/13 (9.8%)
Perihilar region	49 (34.8%)/29 (21.9%)
Common bile duct	84 (59.6%)/82 (62.2%)
Pancreas	4 (2.8%)/8 (6.1%)
Dilatation +/−	46 (16.8%)/227 (83.2%)
CT/USG scan findings	
Could not be obtained	56 (20.5%)
Mass not seen	144 (52.8%)
Mass <10 mm	2 (0.7%)
10–30 mm	23 (8.4%)
Mass >30 mm	48 (17.6%)
Laboratory findings	
Glucose (mg/dL)	104 ± 61.2
ALT (U/L)	112 (7–4660)
AST (U/L)	87.2 (10–2225)
GGT (U/L)	239 (9–2465)
ALP (U/L)	282 (2–3217)
Total bilirubin (mg/dL)	5.7 ± 6.4
Tumor markers	
AFP (U/mL)	1.8 (0.1–1852)
CA-19.9 (U/mL)	64 (1–2085)
CA125 (ng/mL)	20.5 (0.1–1000)
CEA (ng/mL)	2.5 (1–577)

Values are presented as number (%), mean ± SD or median (range).

^∗^3 colon cancer, 1 breast cancer, 1 ovary cancer, and 1 lung cancer.

^∗∗^2 Mirizzi syndrome, 2 choledochal cysts, 2 Oddi Sphincter spasm type 1, and 2, Eisonophilic cholangitis.

**Table 2:** Diagnostic performance of CA19-9, CA 125, brush cytology, and combination of three methods.

	Marker value	Sensitivity %	Specificity %	PPV %	NPV %	Accuracy %
Brush cytology		62.4	97.7	96.7	70.9	79.4
CA-19.9 (U/mL)	72.5	73.8	79.5	79.4	73.9	76.5
CA-125 (ng/mL)	17.5	74.4	61.5	68.1	68.6	68.2
Combination of the three methods^*^		94.1	54.5	80	82.8	80.6

PPV: positive predictive value, NPV: negative predictive value.

^∗^If at least one of the following is positive: brush cytology, CA-19.9, or CA-125.

**Table 3:** The diagnostic success of brush cytology according to stricture location.

Duct^*^	Sensitivity %	Specificity %	PPV %	NPV %	Accuracy %
Intrahepatic	75	100	100	92.9	94.1
Perihilar	69.4	93.1	94.4	64.3	78.2
Common bile duct	57.1	98.8	98	69.2	77.7
Pancreas	%75	100	100	88.9	91.6

NPV: negative predictive value; PPV: positive predictive value.

^∗^No statistically significant difference was found between bile duct and pancreatic duct (*P* = 0.18) or between specific ducts (*P* = 0.415).

**Table 4:** Independent predictors of positive brush cytology.

Variable	*P* value	Odds ratio (95% cl)
Age	0.034	1.025 (1.002–1.04)
Dilatation before sampling	0.006	3.03 (1.5–6.07)
CT/USG scan findings		
Mass <10 mm		1
10–30 mm	0.006	1.7 (1.2–5.7)
>30 mm	0.001	15.04 (4.1–54)
CA-19.9	0.001	1.002 (1.001–1.002)
ALT	0.003	1.004 (1.001–1.007)
Total bilirubin	0.002	1.11 (1.04–1.19)

**Table 5:** Tumor markers in malignant and benign strictures.

Variable	Malign	Benign	*P* value
AFP (U/mL)	2.1 (0.1–1852)	1.6 (0.1–30)	0.002
CA-19.9 (U/mL)	215 (1–2083)	27.3 (1–2085)	0.001
CA 125 (ng/mL)	29 (0.5–528)	15 (0.6–1000)	0.038
CEA (ng/mL)	3 (0.1–577)	2 (0.1–76)	0.001

Values are presented as median (range).
